# Consumer Survey on the Experience of Clozapine Treatment and Monitoring Process in an Australian Community Mental Health Service

**DOI:** 10.1192/bjo.2024.363

**Published:** 2024-08-01

**Authors:** Tharushi Fernando, Ritesh Bhandarkar

**Affiliations:** Monash Health, Melbourne, Australia

## Abstract

**Aims:**

Clozapine is a well-established and widely practiced treatment for treatment-resistant schizophrenia. Due to its significant side effect profile, it requires intense monitoring, including monthly blood tests and medical reviews. A patient's attitude towards clozapine can impact compliance with treatment and its monitoring process. This survey intended to identify the community mental health patients' perception of the clozapine treatment and its monitoring process and to help improve current practices of the service.

**Methods:**

A structured survey with 17 questions was administered to patients registered at the community clozapine clinic via face-to-face or phone conversation at an Australian Community Mental Health Service by the principal researcher and clozapine coordinators.

**Results:**

17 out of 25 eligible patients (68%) participated; the mean age was 39.7 years. There were nine female and eight male participants. 94% of patients were on clozapine for more than one year. 70.5% agreed that clozapine helped to improve mental health, and they understand clozapine side effects and monitoring process. 76.5% agreed that the treating team provided psychoeducation. Seven participants reported clozapine improved side effects compared with previous medications. Three disagreed that clozapine improved side effects, and six remained neutral. Hypersalivation (35.2%), constipation (23.5%) and weight gain (17.6%) were identified as the worst side effects. Nine (52.9%) participants reported that they make healthy life choices. Factors affecting motivation for a healthy lifestyle are mental health symptoms (47%), finances (47%) and physical health well-being (52.9%). Only 35% identified motivation from others as necessary for a healthy lifestyle. Fatigue/poor motivation (47%) and mental health (35.2%) prevent them from making healthy choices. Side effects and finances equally (23.5%) impact healthy choices. Eleven participants (64.7%) felt clozapine monitoring was a positive experience, and 88.2% felt they had enough support during the clozapine monitoring process and were adequately informed about their treatment plan. Two participants disagreed that they were informed of their treatment plan. The majority (82.3%) said no change was needed in the monthly medical officer-led clozapine clinic or six-monthly psychiatrist-led clozapine clinics. Text messages (88.2%) and phone conversations (47%) were the most preferred method for treating team communication about treatment.

**Conclusion:**

The majority of patients identified that clozapine helped to improve mental health, and the monitoring process was a positive experience. Most participants were aware of clozapine and its monitoring process. Psychosocial support will be essential to improve quality of life and might improve the negative perception of clozapine's side effects.

